# Detection of an internal density change in an anthropomorphic head phantom via tracking of charged nuclear fragments in carbon‐ion radiotherapy

**DOI:** 10.1002/mp.17590

**Published:** 2024-12-23

**Authors:** Luisa Schweins, Rebekka Kirchgässner, Pamela Ochoa‐Parra, Marcus Winter, Semi Harrabi, Andrea Mairani, Oliver Jäkel, Jürgen Debus, Mária Martišíková, Laurent Kelleter

**Affiliations:** ^1^ Heidelberg Institute for Radiation Oncology (HIRO) and National Center for Radiation Research in oncology (NCRO) Heidelberg Germany; ^2^ Division of Medical Physics in Radiation Oncology German Cancer Research Center (DKFZ) Heidelberg Germany; ^3^ Department of Physics and Astronomy Heidelberg University Heidelberg Germany; ^4^ Department of Physics Karlsruhe Institute of Technology (KIT) Karlsruhe Germany; ^5^ Department of Radiation Oncology Heidelberg University Hospital Heidelberg Ion‐Beam Therapy Center (HIT) Heidelberg Germany; ^6^ Department of Radiation Oncology Heidelberg University Hospital Heidelberg Germany; ^7^ National Center of Tumor Diseases (NCT) Heidelberg a partnership between DKFZ and University Medical Center Heidelberg Heidelberg Germany; ^8^ Clinical Cooperation Unit Radiation Oncology German Cancer Research Center (DKFZ) Heidelberg Germany

**Keywords:** carbon‐ion‐beam range monitoring, secondary ions, Timepix3

## Abstract

**Background:**

Carbon‐ion radiotherapy provides steep dose gradients that allow the simultaneous application of high tumor doses as well as the sparing of healthy tissue and radio‐sensitive organs. However, even small anatomical changes may have a severe impact on the dose distribution because of the finite range of ion beams.

**Purpose:**

An in‐vivo monitoring method based on secondary‐ion emission could potentially provide feedback about the patient anatomy and thus the treatment quality. This work aims to prove that a clinically relevant anatomical change in an anthropomorphic head phantom may be detected via charged‐fragment tracking during a treatment fraction.

**Methods:**

A clinically representative carbon‐ion treatment plan was created for a skull‐base tumor in an anthropomorphic head phantom. In order to imitate an inter‐fractional anatomical change — for example, through tissue swelling or mucous accumulation — a piece of silicone was inserted into the nasopharynx. Fragment distributions with and without the silicone insert were subsequently acquired with a mini‐tracker made of four hybrid silicon pixel detectors. Experimental irradiations were carried out at the Heidelberg Ion Beam Therapy Centre (HIT, Germany). FLUKA Monte Carlo simulations were performed to support the interpretation of the experimental results.

**Results:**

It was found that the silicone causes a significant change in the fragment emission that was clearly distinguishable from statistical fluctuations and setup uncertainties. Two regions of fragment loss were observed upstream and downstream of the silicone with similar amplitude in both the measurement and the simulation. Monte Carlo simulations showed that the observed signature is a consequence of a complex interplay of fragment production, scattering, and absorption.

**Conclusions:**

Carbon‐ion therapy monitoring with charged nuclear fragments was shown to be capable of detecting clinically relevant density changes in an anthropomorphic head phantom under realistic clinic‐like conditions. The complexity of the observed signal requires the development of advanced analysis techniques and underscores the importance of Monte Carlo simulations. The findings have strong implications for the ongoing InViMo clinical trial at HIT, which investigates the feasibility of secondary‐ion monitoring for skull‐base cancer patients.

## 1 INTRODUCTION

Radiotherapy is one of the most commonly used and effective cancer treatment modalities.^1^ Compared with conventional x‐ray radiotherapy, carbon‐ion radiotherapy (CIRT) has biological and dosimetric advantages.^2^ Thanks to the Bragg peak, ion beams deposit very little dose downstream of the target volume. Heavier ions such as carbon also provide a sharper lateral penumbra and a higher radiobiological effectiveness (RBE) compared with x‐rays and light ions.^3^ Ultimately, these advantages allow the radio oncologist to prescribe more conformal dose distributions that widen the therapeutic window for cancer patients.^4^

However, even small morphological changes in the path of the carbon ions may have a significant impact on the dose distribution and therefore on the treatment outcome.^5^, ^6^, ^7^ A potential solution could be to perform in‐vivo monitoring of the ion beam in the patient.^8^, ^9^, ^10^ Different monitoring methods have been researched, all of which are based on the detection of secondary radiation such as positron annihilation photons (PET), prompt gammas, and charged nuclear fragments (secondary ions). Secondary radiation always emerges from the patient during irradiation and may carry information about the treatment quality. All three techniques offer the possibility of real‐time treatment monitoring at similar clinical practicality.^8^, ^9^, ^10^

This work focuses on treatment monitoring with charged nuclear fragments, which are created when a primary carbon ion disintegrates into lighter ions in nuclear interactions with the patient tissue. The main principle of the monitoring method is to reconstruct the origins of fragments (fragmentation vertices) for several treatment fractions and draw conclusions about inter‐fractional changes in the patient anatomy from the comparison of fragmentation‐vertex distributions. In comparison to prompt gammas and annihilation gammas, fragments have the important advantage of 100% detection efficiency and virtually zero background radiation. Unlike fragments, gamma‐based in‐vivo monitoring techniques in carbon‐ion therapy therefore, suffer from low count rates and signal‐to‐noise ratios. A disadvantage of fragments is their scattering in the patient tissue as well as the strong forward‐emission which limits the achievable spatial resolution of fragmentation‐vertex measurements.^11^ Recent patient measurements at the CNAO ion‐beam therapy center (Pavia, Italy) showed the enormous potential of in‐vivo monitoring with secondary ions when the emptying of the sinus cavity between treatment fractions of a patient suffering from adenoid cystic carcinoma was detected.^12^

Our research group has been using hybrid semiconducting pixel detectors (Timepix and Timepix3) for the tracking of charged nuclear fragments.^13^, ^14^ In previous studies we proved that large metal and air inserts in the beam path can indeed be detected with our tracking system.^15^, ^16^ Further research showed that this also works for 2 mm‐thin air slabs in an otherwise homogeneous cylindrical plastic head model irradiated with carbon ions under realistic clinical conditions.^17^, ^18^, ^19^ Besides the monitoring of tissue density alterations, our system also proved to be capable of tracking the lateral pencil‐beam position with sub‐millimetre accuracy based on the fragment emission.^20^

This work expands upon the previously used simplistic plastic head models by attempting to monitor a realistic case of a density change inside an anthropomorphic head phantom. The introduced density change constitutes of a piece of silicone that was inserted in the nasopharynx of the head phantom. In a CIRT cancer patient, the nasopharynx may be subject to density changes for example, through tissue swelling, tumor volume change, and mucous accumulation. As such, a filling of the nasopharynx with tissue‐density‐equivalent material is a realistic and relevant monitoring goal. Measurements were carried out at the Heidelberg Ion Beam Therapy Center (HIT) using a clinically realistic carbon‐ion treatment plan for a target volume at the base of skull. Moreover, Monte Carlo simulations were implemented to perform an in‐depth analysis of the signal observed in the fragment distributions.^21^ This investigation forms part of the preparatory work for the InViMo (in‐vivo monitoring) prospective clinical trial at HIT, which aims to test contactless fragment‐based monitoring for base‐of‐skull cancer patients.^22^

## 2 MATERIALS AND METHODS

### 2.1 Measurements

#### 2.1.1 Heidelberg ion beam therapy center

All measurements presented in this work were carried out at the HIT (Heidelberg, Germany).^23^ HIT has been treating cancer patients with protons and carbon ions since 2009 as well as helium ions since 2021.^24^, ^25^ There are three fixed horizontal beam lines — two for patient treatments and one for research — as well as an ion‐beam gantry. Treatments are delivered using raster pencil‐beam scanning and active energy modulation.^26^ A ripple filter broadens the carbon‐ion Bragg peak and thus reduces the number of energy layers required for a uniform dose distribution.^27^ The beam application and monitoring system (BAMS) in the beam nozzle continuously measures the pencil‐beam position and intensity. For that it uses two multi‐wire proportional chambers (MWPC) and three ionization chambers (IC). Those data are saved and used in this work to recover the fragment origins with a mini‐tracker, see section 2.3.

#### 2.1.2 Anthropomorphic head phantom

**FIGURE 1:**
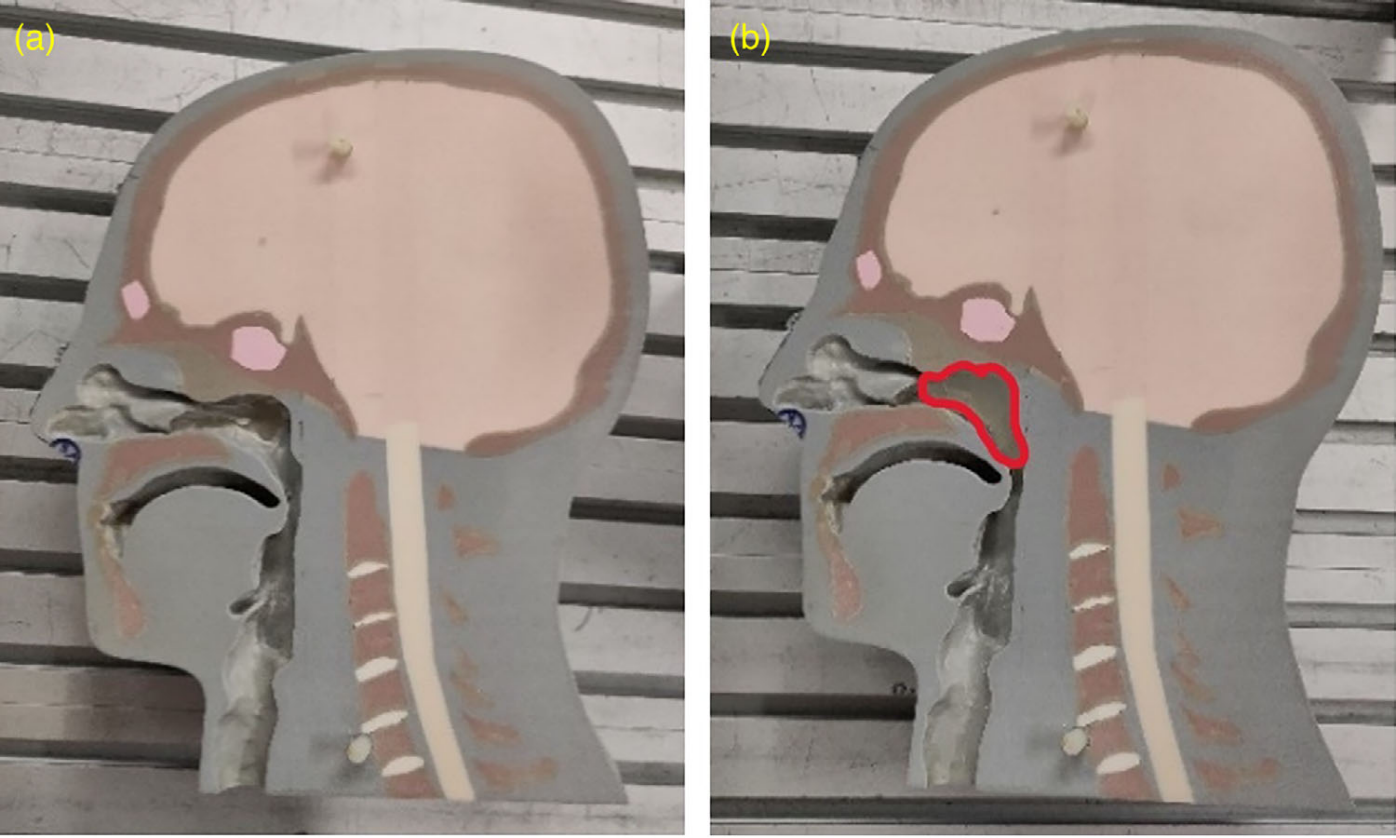
Photograph of the right half of the head phantom (a) without and (b) with silicone insert (highlighted with a red line).

Measurements performed for this work use the anthropomorphic head phantom 731‐HN (Computerized Imaging Reference Systems, Norfolk, VA 23513 United States). The phantom was designed to reproduce the human anatomy of the head as well as the Hounsfield units as measured by a CT scanner and the stopping power of ion beams. It is composed of four sagittal slabs, which can be disassembled in order to access the air‐filled nasal and oral cavities. Clinical practice at HIT showed that the treatment quality of skull‐base cancer patients can be impaired by density changes in the nasopharynx. These may be caused by mucosa swelling, tumor growth, or the accumulation of saliva and mucous. In order to emulate a severe internal density change in a skull‐base cancer patient, 6g (about 6mL) of soft‐tissue‐equivalent silicone with a mean Hounsfield unit of ‐20 (Troll Factory Rainer Habekost e.K., Riede, Germany) was introduced in the nasopharynx. Two CT scans of the head phantom with (follow‐up CT) and without (planning CT) silicone insert were acquired on a SOMATOM Sensation Open CT scanner (Siemens Healthineers AG, Erlangen, Germany) at Heidelberg University Hospital following the default clinical protocols. Photographs of the right half of the head phantom with and without silicone insert are shown in Figure 1.

#### 2.1.3 Treatment plan

**FIGURE 2:**
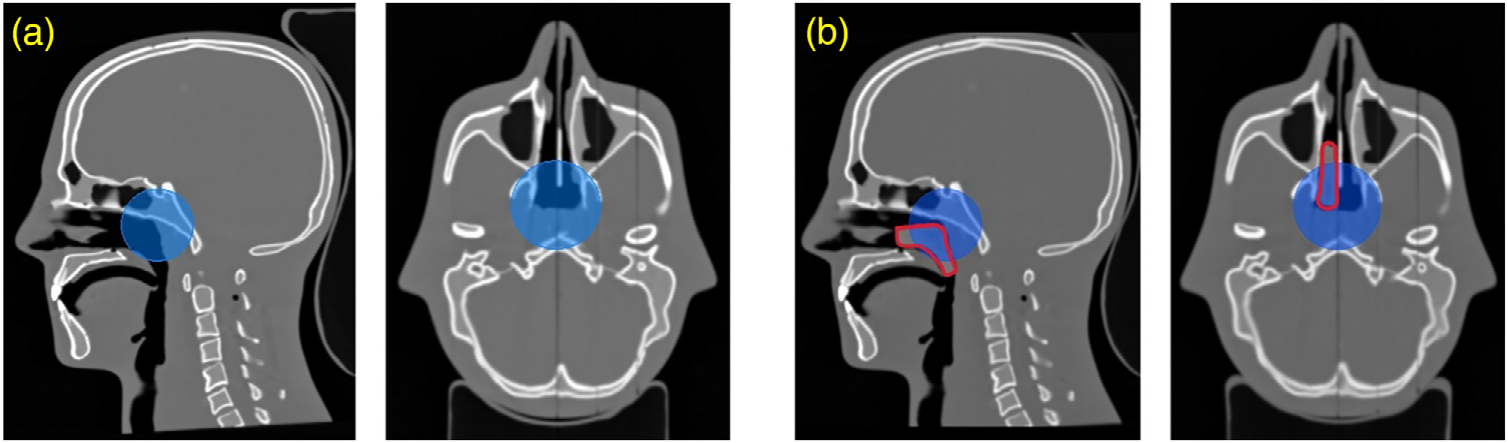
Sagittal and axial CT slices of the head phantom (a) without and (b) with silicone insert (highlighted with a red line). The spherical PTV (blue area) has a diameter of 46 mm. PTV, planning treatment volume.

A representative carbon‐ion treatment plan was designed in RayStation version 11B (RaySearch Laboratories AB, Stockholm, Sweden) for a virtual target volume near the base of the skull in the empty (no silicone insert) head phantom. The planning treatment volume (PTV) is spherical with a volume of 50mL, which corresponds to a diameter of 46mm. The center of the sphere is defined as the planning isocenter, meaning that the center of the sphere will be aligned with the room isocenter during irradiation. As is common in clinical practice at HIT, two opposing fields (beam directions) were used, with a combined fraction dose of 3Gy(RBE) (prescribed dose of 60Gy(RBE) in 20 fractions). Only one treatment field was monitored in this work in order to reduce the workload and save irradiation time. Due to the two‐field biological optimization, the physical fraction dose of one single field appears non‐uniform. The location of the silicone insert relative to the PTV is shown in Figure 2. Pencil beam energies in the treatment field span from 153 MeV $u^{-1}$ to 250 MeV $u^{-1}$. The PTV is subdivided into 25 energy layers with 5942 pencil beams and a total of 3.08 × 10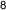 primary carbon ions.

#### 2.1.4 Mini‐tracker

Charged nuclear fragments emerging from a head phantom irradiated with carbon‐ion beams were tracked with a novel mini‐tracker (Advacam s.r.o., Prague, Czech Republic).^28^ It is made of four hybrid silicon pixel detectors (Timepix3), arranged in two detection layers.^29^ The two Timepix3 chips in a layer share a double‐sized silicon sensor of 500μm thickness. Each silicon sensor has an active area of approximately 28 mm × 14 mm. The distance between the two layers is 20.3 mm and the pixel pitch is 55 μm × 55 μm. Timepix 3 employs data‐driven readout with a maximum data rate of 47Mhits $s^{-1}$.^30^ A detection threshold of 3 keV is used to enable noise‐free data acquisition. The timestamp binning of Timepix3 is 1.5625ns, allowing single‐particle detection and efficient coincidence searching in the two detection layers. The default bias voltage of 200V was used for all measurements in this work, resulting in full depletion of the silicon sensor.

#### 2.1.5 Experimental setup

**FIGURE 3:**
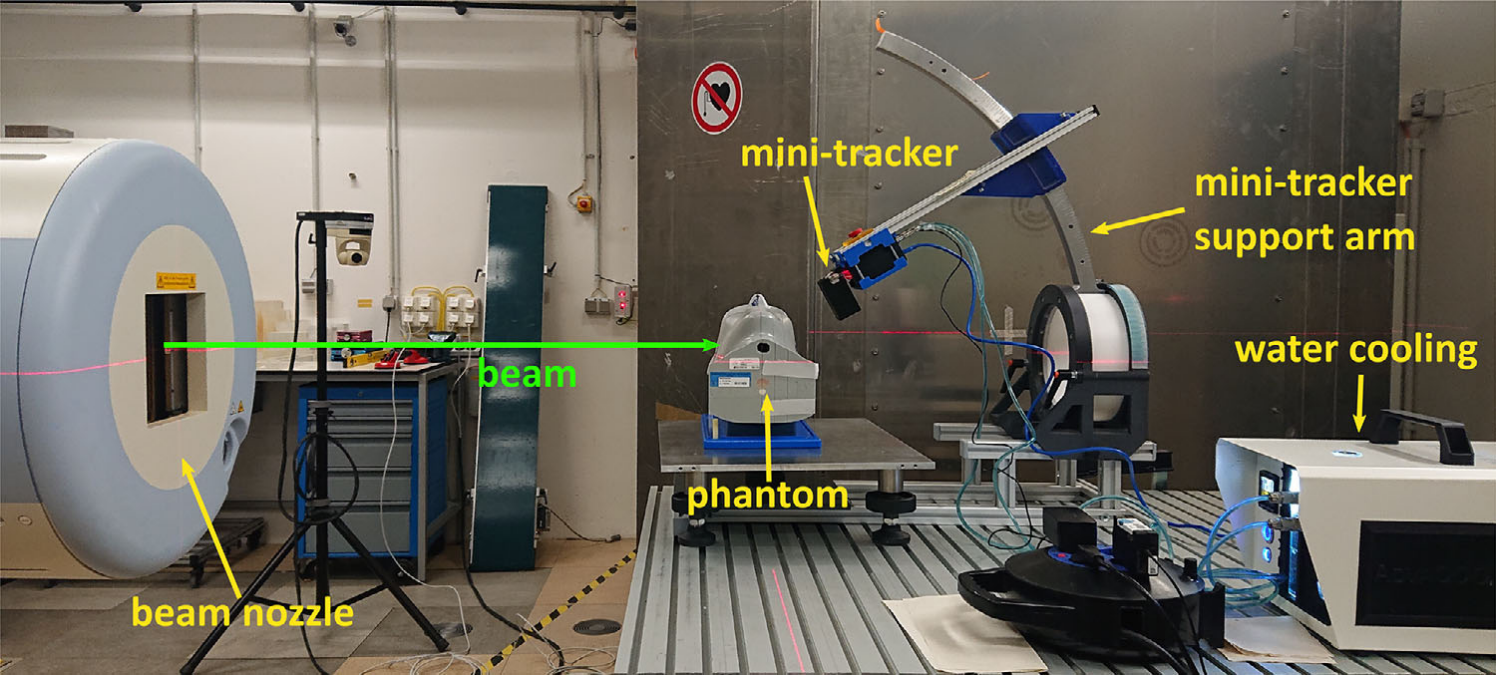
Photograph of the experimental setup at HIT. HIT, Heidelberg Ion Beam Therapy Center.

A photograph of the experimental setup is shown in Figure 3. The phantom is positioned on a headrest in a supine position, just as a human patient would be during radiotherapy. In a clinical patient irradiation, orthogonal x‐rays are used to perform patient positioning with sub‐millimeter accuracy. No orthogonal x‐rays are available at the research beam line at HIT where the presented measurements were performed. The planning isocenter was aligned manually with the room isocenter using the in‐room laser system and markers on the outside of the phantom. No immobilization mask was used in order to facilitate position adjustments.

The mini‐tracker was positioned downstream of the isocenter diagonally above the phantom with the help of a support arm. The support arm has markers that ensure its accurate positioning via the in‐room laser system. The focus line of the mini‐tracker (the line connecting the centers of each detection layer) forms an angle of 30° with the beam axis. The front detection layer is at a distance of 16.7cm to the room isocenter. This mini‐tracker position is motivated by previous studies which showed that it represents an optimal trade‐off between detectability and localization of internal density changes.^17^, ^18^ An active water‐cooling system (Advacam) was used to stabilize the temperature of the mini‐tracker at around 30℃.

The fragment distribution was subsequently measured without (reference measurement) and with a silicone insert in the head phantom. Additionally, the phantom was disassembled and re‐measured without the silicone insert in order to measure the influence of the positioning accuracy on the fragment distribution (reference 1 and 2). Each measurement configuration was repeated seven times (treatment plan was irradiated seven times without moving the mini‐trackers or the phantom) in order to emulate a larger monitoring system with seven mini‐trackers, as is currently being developed.^22^ While the seven repetitions are not a one‐to‐one correspondence to the seven‐tracker system, they allow to approximate the expected data.

### 2.2 Monte Carlo simulations

#### 2.2.1 FLUKA settings and HIT beam line

To supplement the experimental measurements, Monte Carlo simulations were carried out in FLUKA (version 2021.2.5), which has been optimized for use in ion beam therapy.^31^, ^32^, ^33^, ^34^ In this study, in addition to the HADROTHE default settings, the packages for evaporation, coalescence and electromagnetic dissociation were activated as they have been recommended for ion‐beam radiotherapy simulations.^31^ Furthermore, delta rays were deactivated and an energy transport threshold of 20MeV was applied to neutron production in order to reduce the computing time. The activation of both thresholds was found to have a negligible impact (observed changes are smaller than random seed variations) on the simulated fragment distribution. Each simulation of the same setup was repeated seven times with different random seeds in order to emulate the seven measurement repetitions.

In this work, an implementation of the HIT beam line was utilized, which was developed for and used in various previous studies.^35^, ^36^, ^37^ It contains the MWPCs, ICs, and the ripple filter to accurately simulate the transport of ion beams. The FLUKA user routine named *source.f* was adapted to simulate the exact lateral pencil‐beam positions, beam focus sizes, primary carbon‐ion numbers and energies as defined in the treatment plan.

#### 2.2.2 Head phantom and mini‐tracker

**FIGURE 4:**
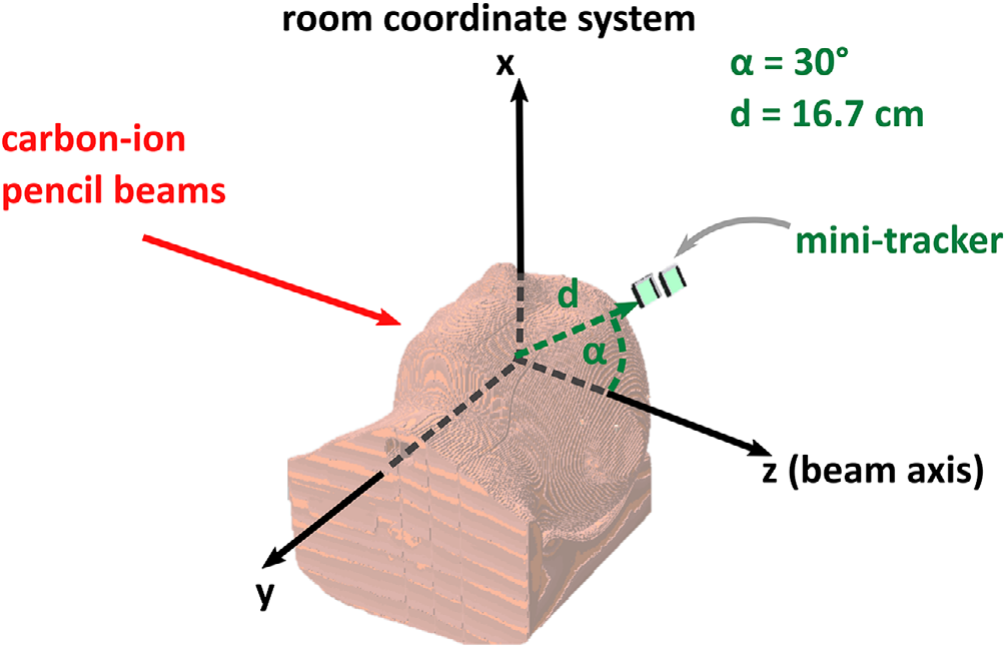
Screenshot showing the CT of the head phantom imported into FLUKA and the sensitive layers of the mini‐tracker, supplemented by the room coordinate system.

In order to accurately simulate the morphology of the phantom, a CT image of the phantom (with and without silicone insert) was imported in FLUKA. For the import and positioning of a CT in FLUKA, FICTION — a python‐based programme developed at HIT — is used to generate the necessary input files.^38^ Hounsfield Units (HU) are grouped into 24 intervals and corresponding material compositions and mass densities are assigned to all voxels accordingly.^39^, ^40^, ^41^ Subsequently, the mass density in the voxel is scaled by the difference between the voxel HU value and the central HU value of the assigned material interval.^42^ A screenshot of the imported head phantom CT in FLUKA with the simulated mini‐tracker is shown in Figure 4.

The sensitive silicon layers (500μm thickness), the 1mm‐thick silicon readout chips, and the aluminium support of the mini‐tracker were realized geometrically accurately in the simulation.^21^ A detailed description and experimental verification of the simulation can be found in Ochoa‐Parra et al. (2024).^21^ In the simulation, a charged particle is considered detected if it deposits more than 3keV (equal to the per‐pixel energy threshold of Timepix3) in each of the two sensitive silicon layers. On top of the fragment interaction points with the mini‐tracker, the locations of the true fragmentation vertices of the tracked fragments are scored. Moreover, the locations of all hadronic inelastic and non‐elastic interactions (HINI) in the phantom were scored. The HINIs include all events that could potentially result in a fragment that is subsequently detected by the mini‐tracker.

### 2.3 Data post‐processing

A detected fragment track is defined by the two interaction points in the front and back detection layers of a mini‐tracker. While the simulation output already contains the 3D locations of the fragment interaction points with the detection layers, the raw data output of Timepix3 requires post‐processing to calculate those interaction points. This is achieved with custom‐developed Matlab 2021 routines (MathWorks, Natick, MA, USA). In the Timepix3 raw data, each pixel signal is characterized by its time of arrival (ToA), time over threshold (ToT, a surrogate of energy deposition) and the 2D position in the silicon sensor. Due to charge‐sharing, coincident neighboring pixel signals need to be combined to form clusters. The timestamp t of a cluster is defined as its earliest pixel signal timestamp. The energy deposition of a cluster is the sum of the individual pixel energy depositions. The 2D position of the cluster is defined as the energy‐weighted mean of the constituent pixel positions.

Fragment tracks are defined by two coincident interaction points in the front and back detection layers of the mini‐tracker. This method strongly suppresses the background from particles other than charged nuclear fragments and thus ensures the purity of the signal. A coincidence window of |Δt|≤75ns was applied. If there are more than two clusters in a coincidence window, the clusters with the smallest |Δt| were matched. Finally, the cluster position in the room coordinate system is calculated based on the known position of the mini‐tracker. A known tilt of the mini‐tracker of 1.6° within its support was taken into account.^28^

In experiment as well as simulation, the reconstructed fragmentation vertices of individual fragments need to be approximated in 3D based on the fragment tracks. A projection algorithm was used to calculate the shortest connection line between a fragment track and its associated pencil‐beam line, as measured by the BAMS (see section 2.1.1). The midpoint on this connection line is defined as the reconstructed fragmentation vertex (FV).^18^ The collection of reconstructed fragmentation vertices is called a fragment distribution. It was shown that this projection method results in a projection uncertainty of 3 –5 mm along the beam axis.^22^

In order to evaluate the signal of a density change in the fragment distribution, two fragment distributions are subtracted. Any significant signal showing up in the fragment distribution difference forms part of the signature that is created by the density change. The aim of this manuscript is to explore and explain this signature based on a comparison of measurement and simulation in the controlled situation of a head phantom.

## 3 RESULTS

### 3.1 Dose distribution

**FIGURE 5:**
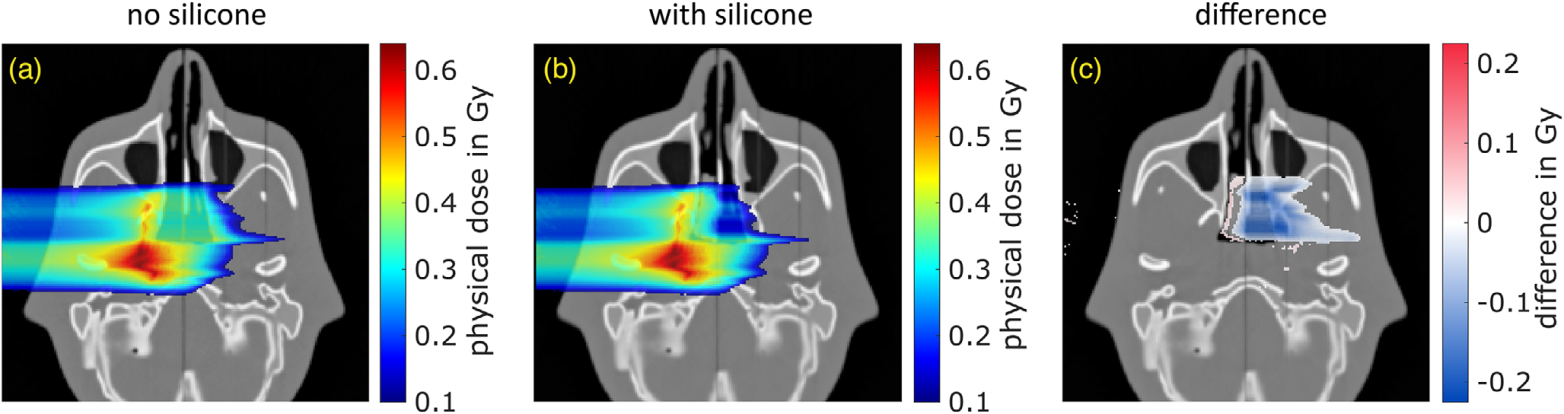
Simulated physical dose distributions corresponding to one fraction of one treatment field (a) without and (b) with silicone insert. The difference between the dose distributions is shown in (c).

The physical dose distribution was simulated in FLUKA using the same treatment plan on both the CT without and with silicone insert. The two simulated dose distributions as well as their difference are shown in Figure 5 with a voxel size of 1 mm × 1 mm × 1 mm. A visualization threshold of 0.1Gy is used for the dose, and of 5% for the dose difference. The latter represents a commonly used dose‐difference threshold at HIT when evaluating the treatment plan quality. The insertion of the silicone leads to an over‐dosage in the right nasopharynx of up to 0.05Gy and an under‐dosage of up to ‐0.22Gy in the left nasopharynx as well as downstream of it.

**FIGURE 6:**
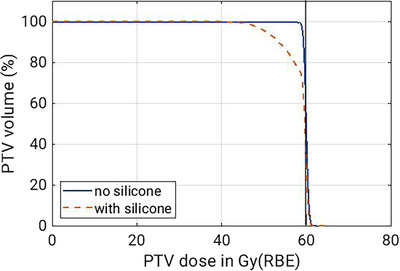
DVH of the PTV for the two‐field treatment plan computed by Raystation. DVH, dose‐volume histogram; PTV, planning treatment volume.

The dose‐volume histogram (DVH) of the entire 30‐fraction two‐field treatment plan was computed in Raystation and is shown in Figure 6. Taking into account the effect of the silicone on both fields, it reflects the total impact of the anatomical change on the treatment quality. The DVH shows a clinically significant under‐dosage in the PTV, with only 90% of the PTV receiving 90% of the prescribed dose.

### 3.2 Fragment distribution

#### 3.2.1 Uncertainty evaluation

**FIGURE 7:**
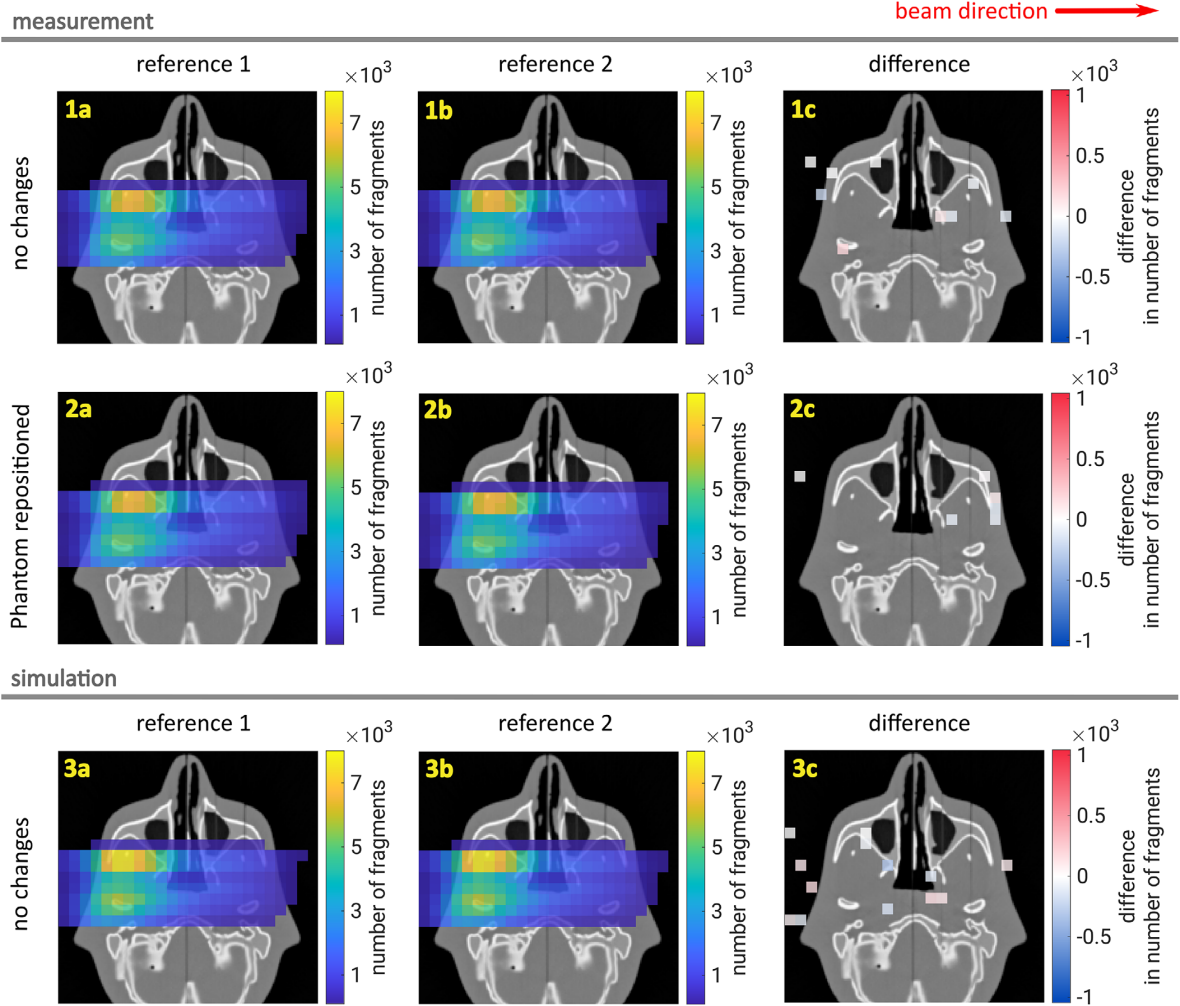
Distributions of measured and simulated reconstructed fragmentation vertices on an axial CT slice that shows the nasopharynx. Column (a) and (b) show two different reference distributions without silicone insert. The difference of the two reference distributions is shown in column (c) in order to perform an uncertainty evaluation.

In order to prove the robustness of the treatment monitoring with charged nuclear fragments, we evaluate here the influence of different uncertainties without the presence of the silicone insert. Again, in order to mimic a larger detection system with seven mini‐trackers, each measurement was repeated seven times, and the fragment distributions were summed up. Figure 7 shows the measured (row (1) and (2)) and simulated (row (3)) reference fragment distributions (columns (a) and (b)) as well as their differences (column (c)) plotted on top of an axial CT slice containing the nasopharynx. A voxel size of 8 mm × 8 mm × 10 mm was used, the latter value defining the slice thickness. A visualization threshold of 100 events per voxel was used. In the difference plots of column (c), an additional threshold of 2σ Poisson uncertainty was applied (requiring a difference exceeding the 95.4% confidence level). This threshold is defined as

(1) $$|N_{2}-N_{1}|\geq 2\sqrt{N_{1}+N_{2}},$$

where $N_{1}$ and $N_{2}$ are the event counts in a voxel of distribution 1 and 2, respectively. The beam direction is from left to right in all plots.

In order to generate the data sets shown in row (1), the measurement was repeated without any changes to the setup. Their difference evaluates the irradiation reproducibility at HIT, the mini‐tracker measurement stability, as well as the statistical fragment counting uncertainty. In between the two reference measurements shown in row (2), the head phantom was taken off the headrest, dis‐assembled into slabs, re‐assembled and re‐positioned on the head‐rest, but without introducing the silicone. This accesses the inevitable but undesired repositioning uncertainty that is linked with the later insertion of the silicone. In row (3), different random seeds were used to generate the two simulated reference data sets, allowing us to quantify the pure statistical fragment counting uncertainty.

Of the 200 voxels with more than 100 counts, 9 are expected to pass the threshold of equation 1 by chance according to Poisson statistics. In Figure 7 we observe 9, 6, and 12 voxels exceeding the threshold in plots (1c), (2c) and (3c), respectively, which is in line with expectations. Hence, it was proved that the analysis method is robust against the explored uncertainties and that the handling of the head phantom can be performed without accidentally introducing a fake signal.

#### 3.2.2 Signature of the internal density change

**FIGURE 8:**
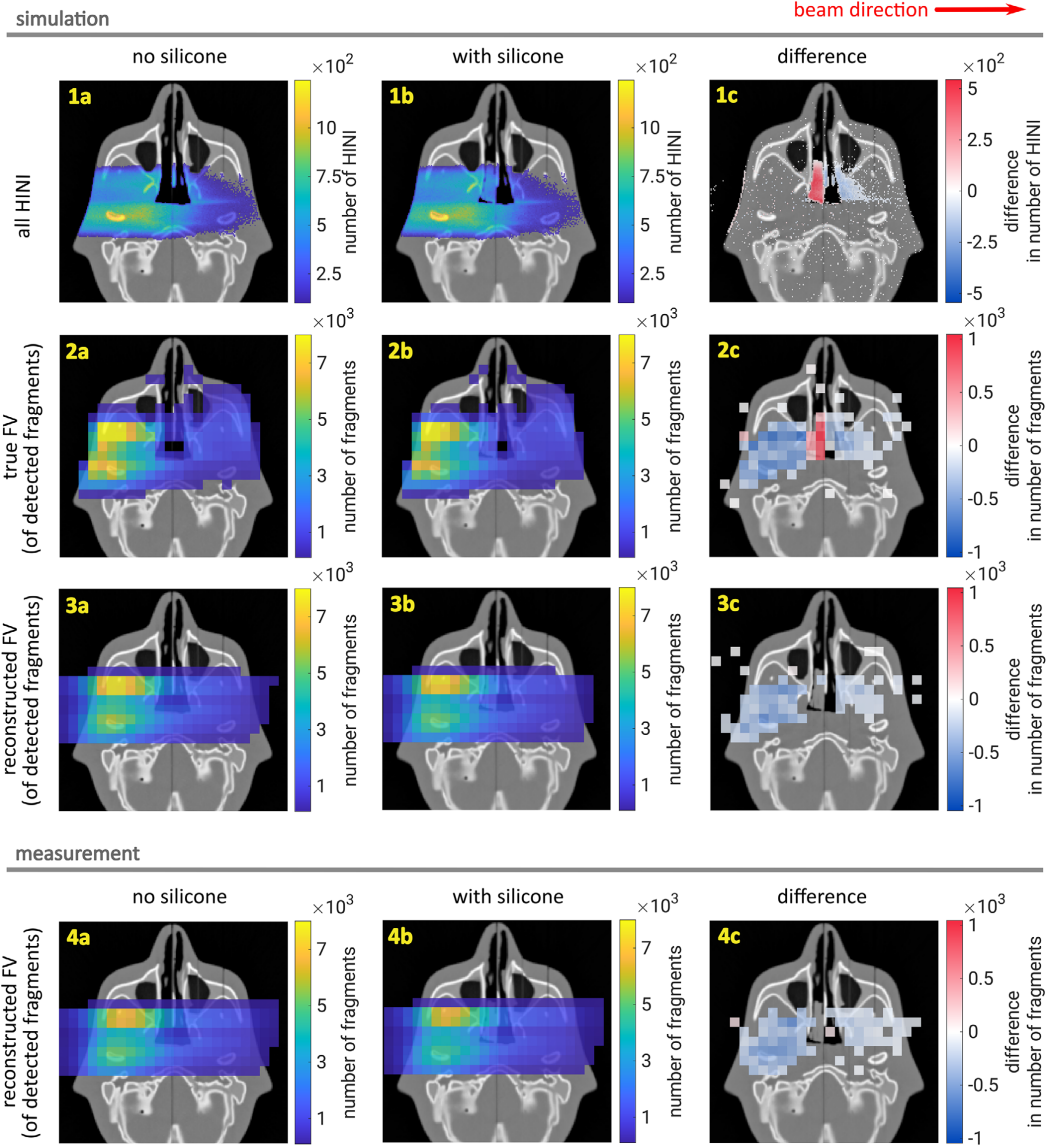
Measured and simulated reconstructed fragmentation vertices (FV) of the detected fragments (row (3) and (4)), complemented by all HINI in the phantom (row (1)) as well as the true fragmentation vertices of the detected fragments (row (2)). The distributions are shown on an axial slice of the planning CT without silicone insert in column (a), with silicone insert on the follow‐up CT in column (b) as well as their difference in column (c). HINI, hadronic inelastic and non‐elastic interactions.

The leading question of this work is whether the dose change caused by the silicone insert can be linked to a signal in the fragment distribution. For that, we compare the 2D distributions of the reconstructed FVs (measurement and simulation), the true FVs (simulation only) and the HINI events (simulation only). The data are visualized in Figure 8 on top of an axial CT slice that includes the nasopharynx. All plots within the same row use the same color axis scale for easier comparison.

The first row of Figure 8 shows the simulated HINI distribution, with a voxel size of 1 mm × 1 mm × 1 mm along the *x*, *y* and *z*‐axes, respectively. In the second row, the simulated true FVs of the detected fragments are visualised. The distributions of the reconstructed FVs are displayed in the third (simulated) and fourth (measured) rows. Rows (2–4) use a voxel size of 8 mm × 8 mm × 10 mm. Column (a) shows the results without silicone insert (planning CT). Column (b) shows the results with silicone insert on the follow‐up CT. The difference between the distributions of columns (a) and (b) is presented in column (c). The same visualization thresholds and color scales were used as in Figure 7.

As explained above, row (1) of Figure 8 includes all hadronic interactions that occur in the head phantom. It can be observed in the bottom left corner of plots (1a) and (1b) of Figure 8 that most hadronic interactions occur where the largest number of primary carbon ions meets the densest tissue (bone). In plot (1c), the influence of the silicone is clearly visible, leading to an increased number of hadronic interactions in the nasopharynx. Furthermore, a reduction of HINI events downstream of the silicone is observed in plot (1c), because primary carbon ions are stopped in the silicone.

The distribution of true FVs shown in row (2) only considers those HINI events that produce a fragment which is subsequently detected by the mini‐tracker. The distribution of true FVs allows the evaluation of the heterogeneous detection probability of fragments generated in the phantom. Instead of being detected in the mini‐tracker, a fragment may be re‐absorbed in the phantom or miss the mini‐tracker, as it covers only a small solid angle of approximately 1.4 × 10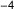 sr. The detection probability depends on fragmentation location because the fragment emission is strongly forward‐peaked and decreases exponentially with increasing angle to the beam axis.^11^ An increased detection probability leads to the appearance of a local fragment emission maximum in the top left corner of the head phantom, upstream of the nasopharynx (plots (2a) and (2b)). The increased fragment production in the silicone and the reduced number of events downstream of the silicone observed in plot (1c) are also visible in the distributions of the true FVs in plot (2c). Furthermore, a loss of detected fragments in the region upstream the silicone is evident in plot (2c). This loss is explained by fragment absorption in the introduced silicone.

Finally, the third and fourth rows of Figure 8 show the distributions of the reconstructed FVs. Compared to the true FVs in the second row, the reconstructed FVs introduce the blurring of the distributions due to Multiple Coulomb Scattering (MCS) of the fragments and the spatial resolution of the mini‐tracker. This blurring leads to the suppression of the signal from the silicone at the location of the nasopharynx, which has vanished in plots (3c) and (4c).

**FIGURE 9:**
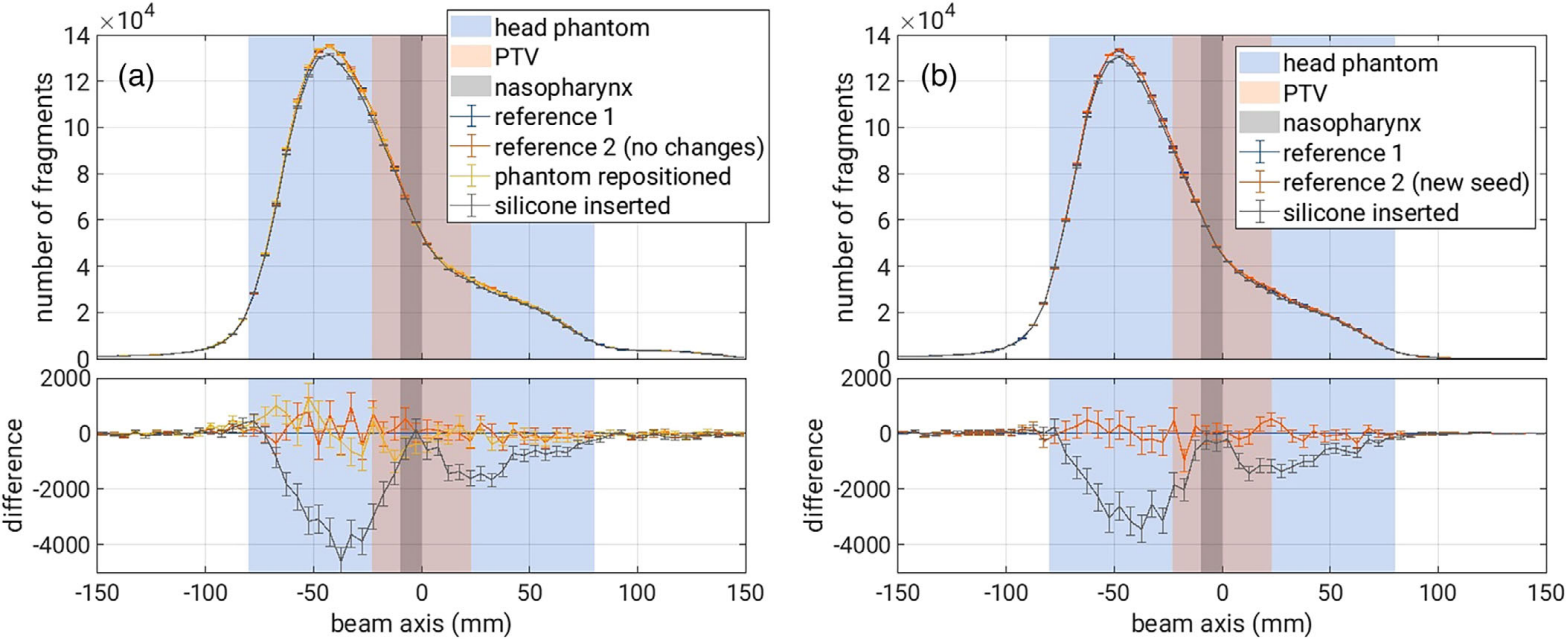
Histograms of FVs along the beam axis for (a) measurement and (b) simulation. The shaded areas highlight the location of the head phantom (blue), the PTV (red) and the nasopharynx (grey) where the silicone was inserted. FV, fragmentation vertex; PTV, planning treatment volume.

Figure 9 shows the depth‐fragment distributions of (a) measurement and (b) simulation in the top panels and the difference to the reference in the bottom panels. The data data is integrated in the transverse plane and the bin size is 5 mm. It can be seen that the distributions without silicone insert are identical within statistical variation. It is worth noting that the distributions of simulated and measured reconstructed FVs are very similar, both quantitatively in terms of the absolute number of fragments and qualitatively in terms of the observed signatures of the silicone insert. A direct comparison of measurement and simulation is provided in a supplementary Figure S1. The observed relative shift between distributions can be traced back to the absolute positioning uncertainty of the mini‐tracker. In Figure 9, the total number of fragment counts in the reference distributions is $2.22\times 10^{6}$ in measurements and $2.05\times 10^{6}$ in simulations (difference of 6%), showcasing the accuracy of the FLUKA implementation. As already seen in the 2D distributions, the insertion of the silicone causes a reduction of fragment counts before (due to fragment absorption in the silicone) and after (carbon‐ion absorption) the silicone insert. The measured total fragment count reduction is −33000 before and −16000 after the silicone. In simulations, the reduction is −28000 and −13000, respectively. Although the shape of the reduction is modelled well in FLUKA, this represents an underestimation of the fragment count reduction of about 16%.

## 4 DISCUSSION

Due to the inserted silicone, the physical fraction dose was reduced by −0.22Gy in distal parts of the PTV. As seen in the DVH, 10% of the PTV would have experienced a dose reduction of 6Gy(RBE) over the course of the full treatment. This represents a clinically relevant under‐dosage of the tumor volume and would have required a treatment plan adaption if it were detected over the course of a carbon‐ion treatment at HIT.

In our most recent work, we managed to detect cylindrical air cavities in a plastic head model down to a size of 10 mm diameter and 2 mm thickness.^19^ However, previous studies of secondary‐ion monitoring have shown that the monitoring sensitivity decreases with increasing depth: the shallower the density change, the easier it is detected.^12^, ^18^ This is because the number of detected fragments decreases with the penetration depth. Moreover, the energy of the primary ion as well as the fragment decreases with increasing depth, which leads to increased scattering and therefore a worse spatial resolution of the fragment origin. Thus, even though the introduced density change is comparatively large by volume and mass, its location inside the PTV at the center of the skull makes it a particularly challenging monitoring case.

Nevertheless, the introduced density change in the nasopharynx resulted in a significant change of the measured and simulated fragment distributions. It can be concluded that the presence of a significant dose change can indeed be detected with secondary‐ion monitoring. The observed signature agreed very well between measurement and simulation, with an integral fragment count difference of 6%. This remaining difference may be attributed to positioning uncertainties of the mini‐tracker and the phantom, as well as uncertainties in the nuclear cross section tables utilized in FLUKA.

However, the signature of the density change in the fragment distribution was found to be complex and not immediately linked to an increase of fragments at the location of the increased density, as could be naively expected. Monte Carlo simulations revealed that while there is indeed an increase of produced fragments at the location of the introduced silicone, an interplay of particle absorption and scattering leads to the much more complicated observed signature. This complexity poses a significant challenge to the interpretation of fragment distributions.

The strong qualitative and quantitative agreement of measurement and Monte Carlo simulation suggests that the observed signature was correctly measured and accurately modeled. Conversely, it indicates that FLUKA can be used for in‐silico studies to retrospectively investigate the benefit of fragment monitoring on historic patient data. As a result, a prospective clinical trial with skull‐base cancer patients is now under way at HIT.

The future will benefit from more sophisticated analysis strategies in order to extract the full information contained in the observed fragment distribution. A major challenge will be to derive with high confidence a specific anatomical alteration from an observed signal between two measured fragment distributions. For example, one could attempt a deconvolution of the reconstructed fragment distribution from the spatial resolution in order to recover the distribution of true fragmentation vertices. The convolution kernels could be derived from Monte Carlo simulations of the same patient. Alternatively, a neural network trained on an extensive simulation library of possible density changes and their fragment‐emission signatures could allow patterns observed in patients to be recognized and identified. Testing the application of various statistical tests may also improve the specificity of this new in‐vivo monitoring method, as has been explored in previous studies.^12^, ^17^

## 5 SUMMARY AND CONCLUSION

This manuscript presents the measurement and simulation of the charged nuclear fragment emission from an anthropomorphic head phantom during carbon‐ion radiotherapy. The aim is to study the fragment signal of an internal density change that would cause a clinically relevant change of the planned dose distribution. Aiming at re‐enacting a realistic clinical patient treatment, a representative treatment plan was designed for a target volume around the base of skull. In order to introduce an inter‐fractional density change, a piece of silicone was inserted in the nasopharynx of the head phantom, mimicking a case of mucous accumulation or tissue swelling. The fragment emission during carbon‐ion radiotherapy was consequently measured with a novel double‐sized Timepix3‐based mini‐tracker at the HIT as well as simulated in the Monte Carlo code FLUKA.

The acquired distributions of the reconstructed fragmentation vertices were analyzed in 3D. Although density changes deeply seated in the patient are the most challenging case to be identified with monitoring methods based on charged nuclear fragments, the cavity filling was indeed detected reliably by our monitoring system. The silicone insert resulted in significant fragment losses upstream and downstream of the nasopharynx, which were clearly distinguishable from statistical fluctuations and setup positioning uncertainties. These findings were confirmed by the FLUKA simulation. Furthermore, the simulation showed that the density change creates a complex interplay of fragment production, scattering, and absorption effects in the phantom. In particular, the limited spatial resolution leads to the suppression of the initially increased fragment production signal at the location of the silicone insert.

Our work shows that it may be challenging to draw straightforward conclusions about internal anatomical changes from inter‐fractional differences between fragment distributions. Advanced data analysis methods will need to be developed in order to access the full potential of this treatment monitoring method. FLUKA‐based Monte Carlo simulations were demonstrated to be a powerful tool for the interpretation of fragment distributions and the conducting of retrospective in‐silico patient studies. Meanwhile, the promising results led to the start of a prospective clinical trial with skull‐base cancer patients at the HIT.

## CONFLICT OF INTEREST STATEMENT

The authors declare no conflicts of interest.

## Supporting information

Figure S1. Comparison of the reference histograms of FVs along the beam axis for measurement and simulation. The shaded areas highlight the location of the head phantom (blue), the PTV (red) and the nasopharynx (grey) where the silicone was inserted. The relative shift is explained by the absolute positioning uncertainty of the mini‐tracker. FV, fragmentation vertex; PTV, planning treatment volume.
