# Supplementary figures and images for: Detection of an internal density change in an anthropomorphic head phantom via tracking of charged nuclear fragments in carbon‐ion radiotherapy
Source: Med Phys. 2024 Dec 23;52(4):2399–411. doi: 10.1002/mp.17590 (PMC11972041; doi:10.1002/mp.17590)

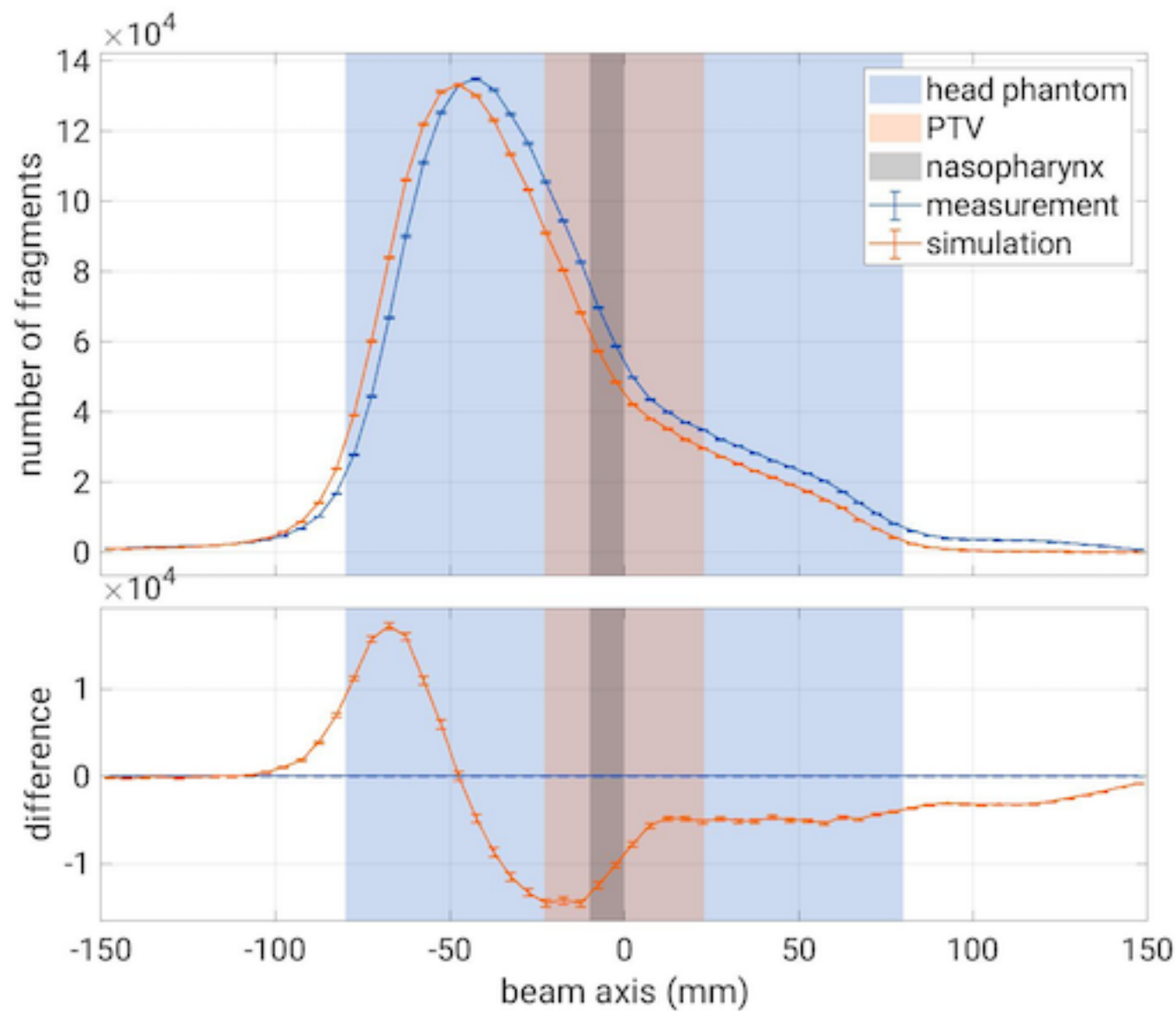

Supplement: Supplementary file 1 — Figure S1. Comparison of the reference histograms of FVs along the beam axis for measurement and simulation. The shaded areas highlight the location of the head phantom (blue), the PTV (red) and the nasopharynx (grey) where the silicone was inserted. The relative shift is explained by the absolute positioning uncertainty of the mini‐tracker. FV, fragmentation vertex; PTV, planning treatment volume. [file MP-52-2399-s001.pdf]
